# Oral Microflora and Its Potential Carcinogenic Effect on Oral Squamous Cell Carcinoma: A Systematic Review and Meta-Analysis

**DOI:** 10.7759/cureus.33560

**Published:** 2023-01-09

**Authors:** Mudiyayirakkani Muthusamy, Pratibha Ramani, Reshma Poothakulath Krishnan, Hemashree K, Gheena Sukumaran, Abilasha Ramasubramanian

**Affiliations:** 1 Department of Oral and Maxillofacial Pathology, Saveetha Dental College and Hospitals, Chennai, IND

**Keywords:** fungi, virus, bacteria, oral microflora, oral squamous cell carcinoma

## Abstract

The oral cavity has the second largest and most diverse microflora. A wide variety of bacteria, viruses, and fungi are present in the oral cavity. A significant number of studies have shown the important role of oral microflora in the initiation and pathogenesis of oral squamous cell carcinoma (OSCC). Microorganisms like *Staphylococcus*, *Streptococcus*, *Neisseria*, *Prevotella*, *Fusobacterium*, *Porphyromonas*, Herpes Simplex Virus I (HSV-1), Epstein-Barr Virus (EBV), Human Papilloma Virus (HPV), *Candida* plays an important role in OSCC. Increased microbial load affects tumor initiation and progression through direct effects on the tumor cells and indirectly through manipulation of the immune system. But the mechanisms describing the steps of oral microflora initiating the OSCC remain an enigma. This systematic review aims to understand the potential carcinogenic effect of oral microflora on OSCC. A systematic literature search was done in PubMed and Google Scholar databases, and six studies were obtained, comprising 1267 participants. The incidence was evaluated as an odds ratio (OR) with a 95% confidence interval (95% CI) using review manager 5.2 software. Oral microflora increased 2.10-fold risk of oral squamous cell carcinoma (OR=2.10, 95% CI: 0.76, 5.84, P= 0.15, I^2^=86%, P_h_<0.00001). In our subgroup analysis, there is a significant relation between Fusobacterium and oral squamous cell carcinoma (OR= 4.86, 95% CI: 0.99, 23.82, P=0.05, I^2^=0%, P_h_= 0.84). Individuals with Epstein-Barr Virus infection exhibit increased incidence of oral squamous cell carcinoma (OR= 3.72, 95% CI: 1.97, 7.04, P=<0.0001, I^2^=0%, P_h_= 0.82). The meta-analysis revealed that oral microflora increases the risk of oral squamous cell carcinoma.

## Introduction and background

Oral cancer is the eleventh most common cancer in the world [1]. Its incidence rate is high in southern Asia (India and Sri Lanka), and it is the leading cause of death among men in India and Sri Lanka [2]. In India, oral cancer constitutes about 40% of all cancers, and it is the most prevalent cancer among men and the third most prevalent cancer in women [3]. In India, the age-standardized incidence rate of oral cancer is 12.6 per 100 000 population, and the incidence is increasing rapidly [3]. More than 90% of oral cancers are oral squamous cell carcinoma (OSCC) [4]. It develops in the lips, buccal mucosa, the floor of the mouth, tongue, alveolar ridges, major salivary glands, minor salivary glands, hard palate, and soft palate [5]. Oral squamous cell carcinoma is a multifactorial disease caused by various risk factors like tobacco, alcohol, betel quid, diet and nutrition, radiation, genetic predisposition, mate, occupational factors, and dental factors [5]. At present, several pieces of scientific evidence have been published exploring the link between oral microflora and OSCC [6,7].

Many reports have shown that microorganisms play an important role in carcinogenesis. Evidence describing the role of *Helicobacter pylori* (*H. pylori*) in gastric cancer reported by various researchers led the World Health Organization International Agency for Research on Cancer (IARC) in 1994 to classify *H. pylori* as a definite cause of cancer in human beings [8,9]. Since then, various types of research have been carried out in linking microorganisms and cancer. After the gut, the oral cavity has the second largest and most diverse microbiota. There are around 700 species of bacteria in the oral cavity. A wide range of bacteria, fungi, viruses, and protozoa are being nurtured in the oral cavity. The oral cavity is a complex habitat where microbes colonize the hard surfaces of the teeth and the soft tissues of the oral mucosa [10]. Based on the frequency of the host and diet, response to the change in pH, interactions among the bacteria, and gene mutations, the oral microorganisms show large and rapid changes in composition and activity [11]. These microorganisms are not pathogenic, and they maintain a symbiotic mutual relationship by checking and preventing the entry and adhesion of other pathogenic microorganisms in the oral cavity. But dysbiosis leads to a failure to control pathogenic microorganisms, to a dysregulated inflammatory or immune response against commensals, and as a result, to severe acute and chronic tissue damage. This is considered a hallmark of cancer [12]. Intense interest has been shown by researchers to understand the association between OSCC and bacteria. Bacteria form the majority group of microorganisms present in the oral cavity [13]. Scientific pieces of evidence have shown that OSCC patients have abnormal bacterial flora with noticeable counts of pathogens, and one study has shown that OSCC patients with normal concentrations of microflora have a better prognosis than patients who do not have normal counts [14]. Not only bacterial species like *Staphylococcus*, *Streptococcus*, *Neisseria*, *Fusobacterium*, and *Porphyromonas* but also viral and fungal species like Epstein-Barr Virus (EBV), Herpes Simplex virus-I (HSV-1), Human Papilloma Virus (HPV), *Candida* also play an important role in the initiation and carcinogenesis of OSCC. In this review, we conducted a systematic literature search in PubMed and Google Scholar on this topic, after which a meta-analysis was conducted to determine the potential carcinogenic effect of oral microflora on oral squamous cell carcinoma.

## Review

Methods

The review was registered in Prospero. The registration id is CRD42022370586.

Search strategy

The databases that were used for searching and collecting the articles were PubMed and Google Scholar, dated from its time of inception till December 2020. The search terms that were used to obtain the results were ((((((((((((((oral microflora) OR (microbes)) OR (oral microorganisms)) OR (oral microbiota)) OR (oral microbiome)) OR (oral bacteria)) OR (oral virus)) OR (oral fungi)) OR (periodontal pathogens)) OR (endodontic pathogens)) OR (oral anaerobic bacteria)) OR (oral aerobic bacteria)) OR (oral gram-positive bacteria)) OR (oral gram-negative bacteria)) AND ((((potential carcinogenic effect) OR (tumourigenesis)) OR (carcinogenesis)) ( oral cancer)) OR (head and neck carcinoma)) OR (oncogenesis))) AND (((((((((((((((((((((((((((((((oral microflora oral cancer) OR (oral microflora oral squamous cell carcinoma)) OR (oral microflora oral carcinoma)) OR ( oral microflora head and neck carcinoma)) OR (oral microflora oral squamous cell neoplasm)) OR (oral microflora oral squamous cell malignancy)) OR (oral microflora oral squamous cell carcinoma tongue)) OR (oral microflora oral squamous cell carcinoma buccal mucosa)) OR (oral microflora oral squamous cell carcinoma hard palate)) OR (oral microflora oral squamous cell carcinoma soft palate)) OR (oral microflora oral tumour)) OR (oral microflora oral malignancy)) OR (oral microflora oral neoplasm)) OR (oral microflora alveolar tumour)) OR (oral microflora alveolar carcinoma)) OR (oral microflora alveolar cancer)) OR (oral microflora gingivobuccal tumour)) OR (oral microflora gingivobuccal carcinoma)) OR (oral microflora cheek tumour)) OR (oral microflora cheek cancer)) OR (oral microflora tongue neoplasm)) OR (oral microflora tongue tumour)) OR (oral microflora tongue cancer)) OR (oral microflora head and neck neoplasm)) OR (oral microflora head and neck carcinoma)) OR (oral microflora lingual tumour)) OR (oral microflora lingual carcinoma)) OR (oral microflora lingual cancer)) OR (oral microflora buccal neoplasm)) OR (oral microflora buccal tumour)) OR (oral microflora buccal carcinoma)) OR (oral microflora buccal cancer)) which resulted in 47 articles.

Inclusion and exclusion criteria

Inclusion Criteria

Articles that were published as original research. The diagnosis of oral squamous cell carcinoma was confirmed by pathological examination.

Exclusion Criteria

Reviews, meeting abstracts, animal studies, pilot studies, case reports, case series.

Statistical analysis

Review Manager 5.2 (Rev Man 5.2) was used for the statistical analysis. The heterogeneity between the included studies was calculated by using the I2 test. The random effect model was used to calculate the odds ratio (OR) and 95% confidence interval (CI). A two-tailed p-value of <0.05 was considered statistically significant.

Results

**Figure 1:**
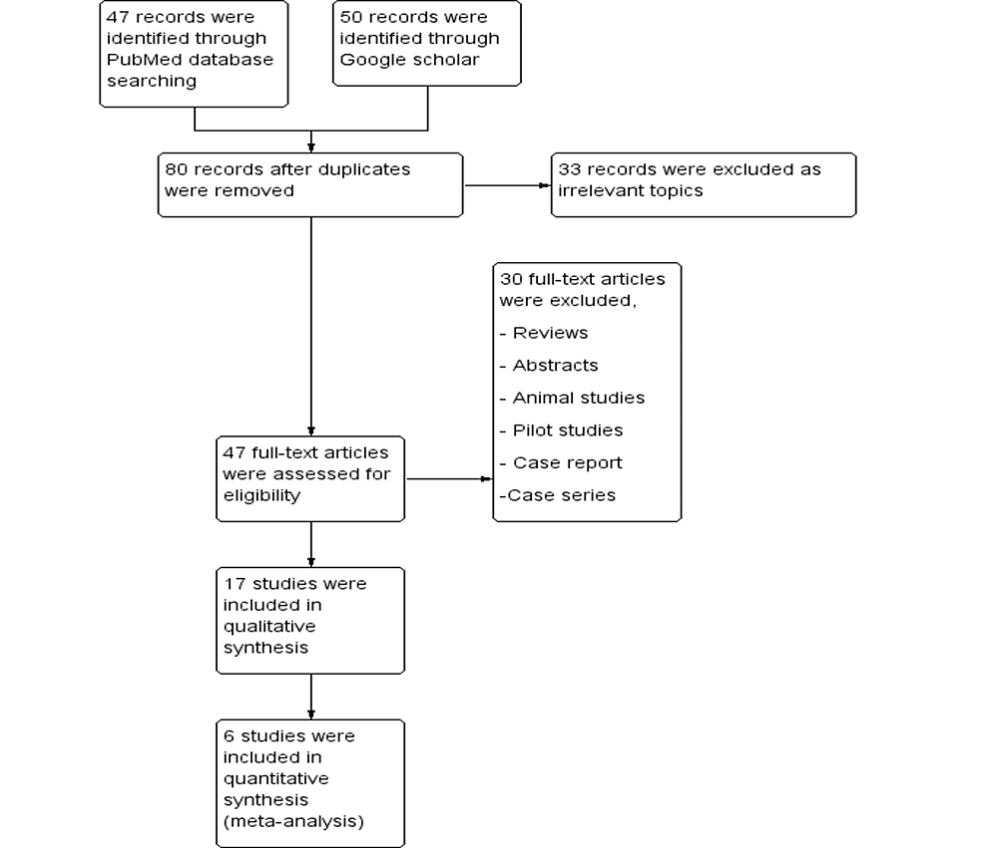
PRISMA flowchart The PRISMA flow chart represents the process of literature search and screening.

The studies included in this article were selected by the process, which is summarized in Figure 1. From the initial 95 articles identified through PubMed and Google Scholar, 78 articles were excluded. The excluded records comprised irrelevant topics, case reports, case series, pilot studies, abstracts, reviews, and animal studies. Seventeen articles were included in the systematic review, from which six studies were included in the meta-analysis that enrolled 1,267 participants. The articles were published from 1988 to 2020 (Table 1). 

**Table 1:** Characteristics of the included studies The table represents the characteristics of the included studies in the meta-analysis, describing the study design, sample size, samples are taken, methods of detection.

S.No	Author	Year	Sample size	Sample	Detection method	Study design
1.	Acharya et al. [15]	2015	N=185 Cases:91 Controls:94	Exfoliated oral cells	Nested PCR	Case-control study
2.	Jain M [16]	2016	N=150 Group I:50 (oral cancer) Group II:50 (pre-cancer) Group III:50 (control)	Blood	PAP technique	Case-control study
3.	Kassim et al. [17]	1988	N=188 Cases:132 Controls:56	Tissue	Herpes Select-1 ELISA	Case-control study
4.	Nagy et al. [18]	1998	N=42 Cases: 21 Controls: 21	Biofilm	Aerobic and anaerobic culture	Case-control study
5.	Sand et al. [19]	2002	N=107 Group 1: 29 (OSCC) Group II: 23 (oral lichen planus) Group III:55 (controls)	Tissue	Nested PCR	Case-control study
6.	Zhang et al. [20]	2020	N=100 Cases: 50 Controls: 50	Tissue	PCR, 16S RNA sequencing.	Case-control study

Interpretation

Association Between Oral Microflora and Oral Squamous Cell Carcinoma

Oral microflora increased 2.10-fold risk of oral squamous cell carcinoma (OR=2.10, 95% CI: 0.76, 5.84, P= 0.15, I2=86%, Ph<0.00001). In our subgroup analysis, there is a significant relation between Fusobacterium and oral squamous cell carcinoma (OR= 4.86, 95% CI: 0.99, 23.82, P=0.05, I2=0%, Ph= 0.84). Individuals with Epstein-Barr Virus (EBV) infection exhibit an increased incidence of oral squamous cell carcinoma (OR= 3.72, 95% CI: 1.97, 7.04, P=<0.0001, I2=0%, Ph= 0.82). However, there was no significant relation between *Streptococcus* (OR= 0.58, 95% CI: 0.23,1.45, P=0.24, I2=19%, Ph=0.27), *Prevotella* (OR=1.67, 95% CI: 0.52,5.41, P=0.39, I2=0%, Ph=0.84), *Neisseria* (OR= 0.75, 95% CI: 0.26,2.15, P=0.59, I2=0%,Ph=0.93%), *Porphyromonas* (OR=2.98, 95% CI: 0.44,20.21, P=0.26, I2=0%, Ph=0.62%), Herpes Simplex Virus-1 (HSV-1) (OR= 18.80, 95% CI: 0.68,516.84, P=0.08, I2=81%,Ph=0.82 as discussed in Table 2.

Heterogeneity and Sensitivity Analysis

There was evidence of heterogeneity in the overall effect of microflora and oral squamous cell carcinoma (I2=86%, Ph<0.00001). In the subgroup, HSV-1 showed significant evidence of heterogeneity (I2= 81%, Ph= 0.02), and the subgroup of *Streptococcus* (I2= 19%, Ph=0.27) showed minimal evidence of heterogeneity, as discussed in Table 2.

**Table 2:** Overall and subgroup analysis The table represents the overall and subgroup analysis of the oral microflora among the selected studies as a result of the meta-analysis.

Variable	Study No.	sample size	OR (95% CI)	P-value	Heterogeneity	
					I^2^	P-value
Overall	6	1,267	2.10[0.76,5.84]	0.15	86%	<0.00001
Bacteria:						
Streptococcus	2	142	0.58[0.23,1.45]	0.24	19%	0.27
Prevotella	2	142	1.67[0.52.5.41]	0.39	0%	0.84
Fusobacterium	2	142	4.86[0.99,23.82]	0.05	0%	0.67
Neisseria	2	142	0.75[0.26,2.15]	0.59	0%	0.93
Porphyromonas	2	142	2.98[0.44,20.21]	0.26	0%	0.62
Virus:						
HSV-1	2	288	18.80[0.68,516.84]	0.08	81%	0.02
Epstein-Barr virus	2	269	3.72[1.97,7.04]	<0.0001	0%	0.82

Discussion

Our meta-analysis included patient-level data on prognostic factors. The subgroup analysis was conducted and analyzed in 1,267 cases based on the characteristics, methodologies, and results of the study. We differentiated the effect of specific oral microorganisms and oral squamous cell carcinoma. From 17 studies, six studies were selected for the meta-analysis.

**Figure 2:**
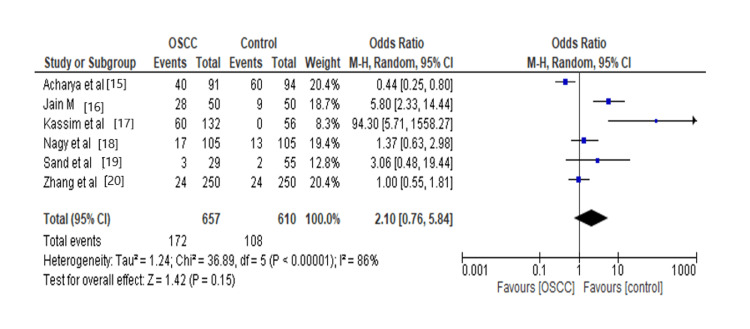
Forest plot: Overall comparison of oral microflora The forest plot represents a meta-analysis of the overall comparison of oral microflora in oral squamous cell carcinoma cases and controls.

Many studies have demonstrated that oral microflora participates in OSCC-promoting pathways. In our meta-analysis, we found that oral microflora increases 2.10 times the risk for oral squamous cell carcinoma, as shown in Figure 2. Some microorganisms are considered key players in the development of OSCC, whereby even in low abundance, their effect is powerful enough to impact the remaining environment [21].

**Figure 3:**
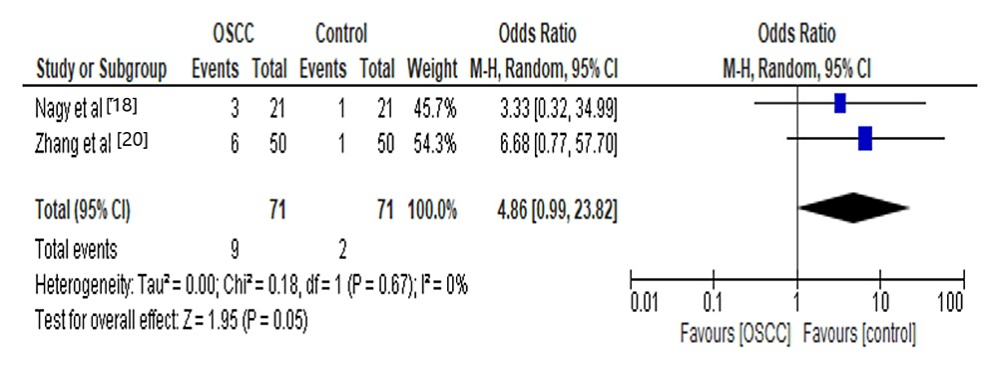
Forest plot: Comparison of Fusobacterium The forest plot represents a meta-analysis showing a comparison of *Fusobacterium* in oral squamous cell carcinoma cases and controls.

In our analysis, we found that *Fusobacterium* increased 4.86 times the risk for OSCC, as shown in Figure 3. The mechanism in OSCC due to the effect of *Fusobacterium* may be because of the production of Matrix Metallo proteinase-13 (MMP) as a result of activation of mitogen-activated protein kinase p38, which leads to Heat shock protein (HSP-27) activation inducing MMP-9 which drives tumor invasion and metastasis [22,23]. *Fusobacterium* synergistically induces the proinflammatory microenvironment along with other microflora through the recruitment of tumor-infiltrating cells, favoring tumorigenesis [24].

**Figure 4:**
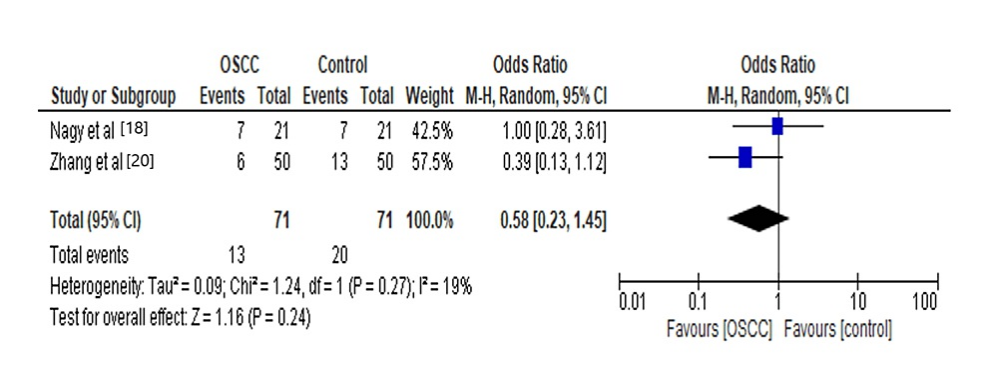
Forest plot: Comparison of Streptococcus in OSCC and controls The forest plot represents a meta-analysis showing a comparison of *Streptococcus* in oral squamous cell carcinoma cases and controls.

Various studies attempted in characterizing the relationship between oral microflora and OSCC. In our analysis, we found that *Streptococcus* species increase 0.58 times the risk for OSCC, as shown in Figure 4. There is increasing evidence that *Streptococcus anginosus* plays an important role in carcinogenesis through the integration of extrinsic *Streptococcus* *anginosus* DNA into the host genome, and another mechanism is a hit-and-run injury to the host genome. *Streptococcus* affects OSCC by producing lactic acid, acetic acid, butyric acid, isobutyric acid, and isovaleric acid, which reduces the environmental pH in OSCC, contributing to the growth and spread of OSCC [25].

**Figure 5:**
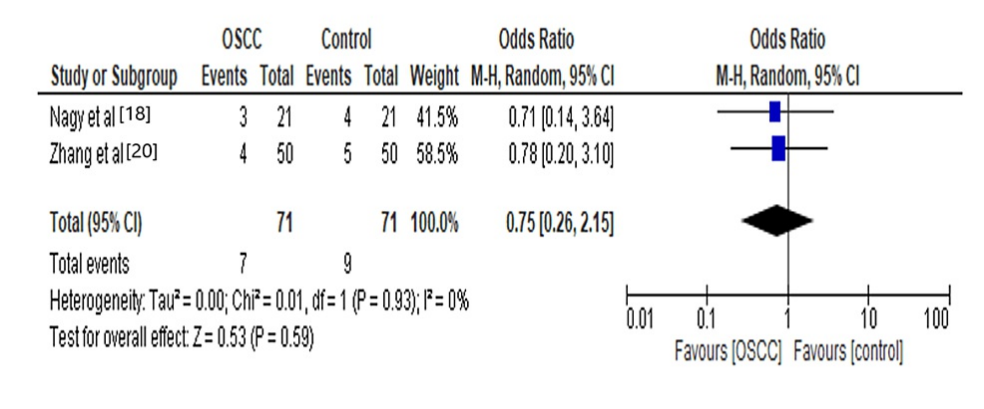
Forest plot: Comparison of Neisseria in OSCC and controls The forest plot represents a meta-analysis showing a comparison of *Neisseria* in oral squamous cell carcinoma cases and controls.

Since changes in the gut microbial composition may contribute to cancer initiation and progression [26]. Oral microbial dysbiosis occurs when the pathogenic bacteria dominates. Oral dysbiosis plays an important role in the manipulation of the host response. It may also be involved in the occurrence and development of OSCC. The results of our analysis showed *Neisseria* increases 0.75 times the risk for OSCC, as shown in Figure 5. An evident reason for carcinogenesis due to *Neisseria* is that it is involved in the significant production of acetaldehyde from ethanol as it has a high alcohol dehydrogenase (ADH) activity [27]. The addition of chemical irritants like alcohol makes the mucosa more sensitive to acetaldehyde produced by *Neisseria* species and thereby increasing the risk of OSCC [28].

**Figure 6:**
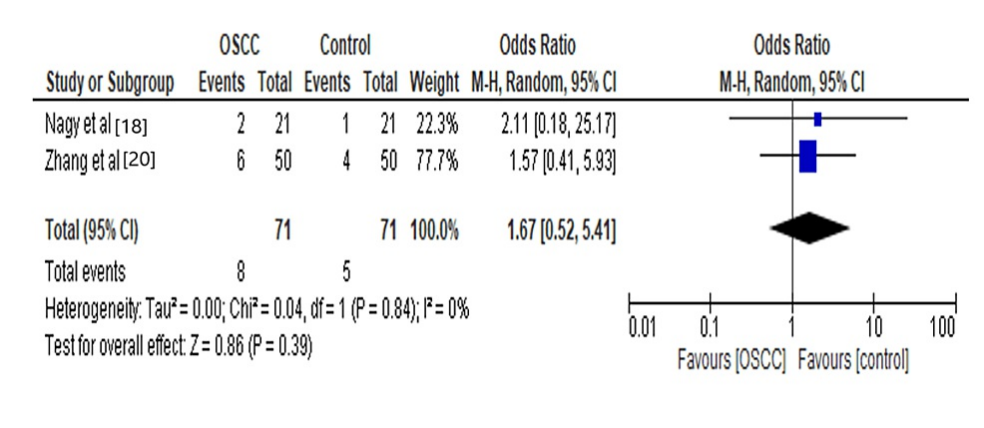
Forest plot: Comparison of Prevotella The forest plot represents a meta-analysis showing a comparison of *Prevotella* in oral squamous cell carcinoma cases and controls.

Bacteria at certain body sites have been believed to be involved in immune modulation and disease development [29]. Our analysis revealed that *Prevotella* increases 1.67 times the risk for OSCC, as shown in Figure 6. A potential reason for the rise of *Prevotella* *melaninogenica* is that alterations in tumor cell receptors could change the adhesion of certain specific bacteria [30]. There are pieces of evidence explaining the effect of *Prevotella* on OSCC by stimulating the production of inflammatory mediators and having harmful effects on fibroblasts, epithelial and endothelial cells, and extracellular matrix components affecting the growth of local concentrations of cytokines like interleukin (IL-6, IL-17), tumor necrosis factor (TNF-α), MMP-8, MMP-9 [31].

**Figure 7:**
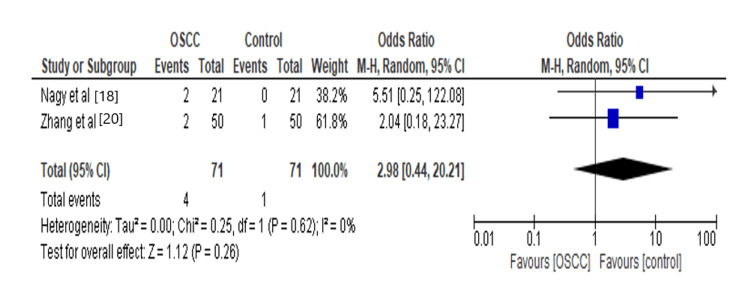
Forest plot: Comparison of Porphyromonas The forest plot represents a meta-analysis showing a comparison of *Porphyromonas* in oral squamous cell carcinoma cases and controls.

Some periodontal pathogens might participate in various pathways to assist OSCC development. In our analysis, we found that *Porphyromonas* increases 2.98 times the risk for OSCC, as shown in Figure 7. It was found that *Porphyromonas gingivalis* (*P. gingivalis*) inhibits macrophages from attacking the Cal-27 cells via membrane-component molecules [32]. Volatile sulfur compounds, like hydrogen sulfide and methyl mercaptan, are produced by *Porphyromonas*. These compounds cause oxidative stress leading to DNA damage in oral calls. Low levels of hydrogen sulfide are sufficient to inhibit the enzyme superoxide dismutase, which is critical in preventing destructive reactive oxygen species (ROS) buildup in human cells, and methyl mercaptan has been implicated in collagen breakdown (type IV), leading to invasion of OSCC into basement membrane [33].

**Figure 8:**
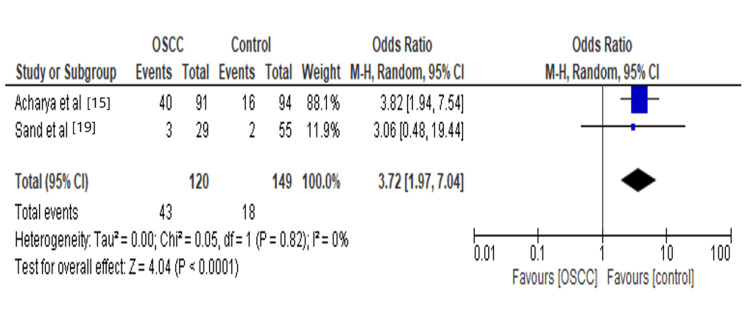
Forest plot: Comparison of EBV The forest plot represents a meta-analysis showing a comparison of Epstein-Barr virus in oral squamous cell carcinoma cases and controls.

Virus plays an important role in carcinogenesis and increases the risk of OSCC. We observed that Epstein-Barr virus (EBV) increases 3.72 times the risk for oral squamous cell carcinoma, as shown in Figure 8. The evident explanation of the mechanism of carcinogenesis is that EBV is latent in malignant conditions, and this latency allows for sustained expression of viral oncogenes while avoiding the immune detection and cytopathic effects from the replicative phase of the viral cell cycle. So, high viral loads are correlated with tumor development [34].

**Figure 9:**
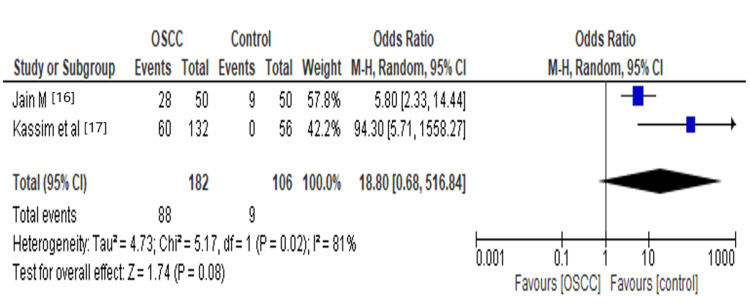
Forest plot: Comparison of HSV-1 The forest plot represents a meta-analysis showing a comparison of Herpes Simplex Virus-1 in oral squamous cell carcinoma cases and controls.

Our study has shown that Herpes simplex virus-1 (HSV-1) increases 18.80 times the risk for OSCC, as shown in Figure 9. A viral protein designated as MUT, which is involved in host cell shut off, is being expressed by Herpes Simplex Virus-1 [35]. HSV-1 induces expression of stress or heat shock proteins which plays an important role in the malignant transformation of the cells [36] It is found that HSV-1 directly transforms the cells to malignant phenotype through mutagenic properties by inducing chromosomal rearrangements.

About six case-control studies subjected to meta-analysis revealed that the oral microflora increases 2.10 times the risk for oral squamous cell carcinoma. The risk notably decreased in patients with good oral hygiene. Alcoholics have an increased risk for OSCC. This is because generally the *Neisseria* species present in our normal microflora have high ADH activity, and upon addition of ethanol by alcohol ingestion, *Neisseria* species will produce a significantly higher amount of acetaldehyde. The accumulation of acetaldehyde produced by *Neisseria* might be sufficient to affect the epithelial cells increasing the risk for OSCC. Habitual drinking provides *Neisseria* a growth advantage over ADH-negative bacteria. Not only alcohol drinking but also cigarette smoking also influenced the bacterial composition of the oral microflora [28]. There are no scientific pieces of evidence on gender differences between the organisms studied. Further researches have to be conducted to note the gender difference between the organisms.

Though the meta-analysis has been done comprehensively, it has its limitations too. The included studies have been conducted in a diverse population, and each study included varied specimens and methods of analyzing the samples leading to varied heterogeneity. Future studies should consider focusing more on the effect of oral microflora on oral squamous cell carcinoma.

## Conclusions

To summarize, various pieces of research on the risk of microorganisms and oral squamous cell carcinoma have illuminated our knowledge of bacterial, viral, and fungal involvement in the carcinogenesis of oral squamous cell carcinoma. However, doubts about the role of microflora in initiation of oral squamous cell carcinoma still remain unclear. Further studies on investigating the role of microflora in the initiation of oral squamous cell carcinoma should be attempted to get a clear idea. It is also important to note that changes in oral microflora in oral squamous cell carcinoma may act as a diagnostic indicator. This meta-analysis suggested that oral microflora increases the risk of oral squamous cell carcinoma, which emphasizes that more focus on research is required in this area which could provide an important clue in the pathogenesis and treatment of OSCC.
